# INTEGRATING AGE-FRIENDLY QUALITY IMPROVEMENT INTO RURAL CLINICAL ROTATION FOR MEDICAL STUDENTS

**DOI:** 10.1093/geroni/igae098.0066

**Published:** 2024-12-31

**Authors:** Keri Christensen, Jennifer Severance, Lesca Hadley

**Affiliations:** University of North Texas Health Science Center, Fort Worth, Texas, United States; University of North Texas Health Science Center, Fort Worth, Texas, United States; University of North Texas Health Science Center, Fort Worth, Texas, United States

## Abstract

Early learning experiences in quality improvement (QI) prepare medical students for practice in complex health systems and for improving processes of care for older adult patients. We developed a curriculum using the Institute for Healthcare Improvement (IHI) Age-Friendly Health Systems 4Ms framework to train third-year medical students (n=75) in geriatrics-focused QI during a twelve-week family medicine clinical rotation in rural communities. Prior to the rotation, students completed asynchronous online training introducing QI models, Plan-Do-Study-Act (PDSA) cycles, and the Age-Friendly 4Ms framework. During the rotation, students met with their preceptor to identify a goal and conduct project activities to enhance Age-Friendly care for older adults within their practice setting. Students also collected information about the local community and patient outcome data related to CMS quality measures. Geriatrics faculty conducted weekly virtual meetings with students to discuss projects and challenges. A final written assignment summarizing their project results, clinical implications and next steps for the primary care setting in a standard abstract format that can be used for a poster presentation at a professional conference. Students (n=71) completed evaluation surveys to rate the usefulness of training and to self-assess knowledge and skills using five-point Likert-type items. Students (n=50) reported increased knowledge and skills in conducting QI activities and improved understanding of the importance of improving processes and patient care. Integrating the Age-Friendly Health Systems framework into QI training can support competency development in system-based practice while disseminating Age-Friendly practices and resources within a health care setting.

